# Serotype distribution, antimicrobial susceptibility, antimicrobial resistance genes and virulence genes of *Salmonella* isolated from a pig slaughterhouse in Yangzhou, China

**DOI:** 10.1186/s13568-019-0936-9

**Published:** 2019-12-28

**Authors:** Quan Li, Jian Yin, Zheng Li, Zewei Li, Yuanzhao Du, Weiwei Guo, Matthew Bellefleur, Shifeng Wang, Huoying Shi

**Affiliations:** 1 College of Veterinary Medicine, Yangzhou University, Yangzhou, 225009 Jiangsu People’s Republic of China; 2 Jiangsu Co-innovation Center for the Prevention and Control of Important Animal Infectious Diseases and Zoonoses, Yangzhou, 225009 China; 3 Yebio Bioengineering Co, Ltd of Qingdao, Qingdao, 266114 China; 4 Department of Infectious Diseases and Immunology, College of Veterinary Medicine, University of Florida, Gainesville, FL 32611-0880 USA; 5 Key Laboratory of Animal Infectious Diseases, Ministry of Agriculture, Yangzhou University, Yangzhou, China; 6 Jiangsu Key Laboratory of Preventive Veterinary Medicine, Yangzhou University, Yangzhou, China

**Keywords:** *Salmonella*, Pig slaughterhouse, Antimicrobial susceptibility, Antimicrobial resistance genes, Virulence genes

## Abstract

*Salmonella* is an important food-borne pathogen associated with public health and high economic losses. To investigate the prevalence and the characteristics of *Salmonella* in a pig slaughterhouse in Yangzhou, a total of 80 *Salmonella* isolates were isolated from 459 (17.43%) samples in 2016–2017. *S.* Derby (35/80, 43.75%) was the most prevalent, followed by *S.* Rissen (16/80, 20.00%) and *S.* Newlands (11/80, 13.75%). The highest rates of susceptibility were observed to cefoxitin (80/80, 100.0%) and amikacin (80/80, 100.0%), followed by aztreonam (79/80, 98.75%) and nitrofurantoin (79/80, 98.75%). The highest resistance rate was detected for tetracycline (65/80, 81.25%), followed by ampicillin (60/80, 75.00%), bactrim (55/80, 68.75%), and sulfisoxazole (54/80, 67.50%). Overall, 91.25% (73/80) of the isolates were resistant to at least one antibiotic, while 71.25% (57/80) of the isolate strains were multidrug resistant in the antimicrobial susceptibility tested. In addition, 86.36% (19/22) of the 22 antimicrobial resistance genes in the isolates were identified. Our data indicated that the resistance to certain antimicrobials was significantly associated, in part, with antimicrobial resistance genes. Furthermore, 81.25% (65/80) isolates harbored the virulence gene of *mogA*, of which 2 *Salmonella* Typhimurium isolates carried the *mogA*, *spvB* and *spvC* virulence genes at the same time. The results showed that swine products in the slaughterhouse were contaminated with multidrug resistant *Salmonella* commonly, especially some isolates carry the *spv* virulence genes. The virulence genes might facilitate the dissemination of the resistance genes to consumers along the production chain, suggesting the importance of controlling *Salmonella* during slaughter for public health.

## Introduction

*Salmonella* has emerged as a major food-borne pathogen associated with breeding industry and public health in many countries (Eurosurveillance editorial team 2012; Majowicz et al. 2010; Kasimoglu Dogru et al. 2010). So far, more than 2600 identified serovars of *Salmonella* have been recorded (Guibourdenche et al. 2010). It is one of the leading causes of human gastroenteritis and causes more than 93.8 million infection cases annually (Majowicz et al. 2010). *Salmonella* has been estimated at least 1 million cases in the USA each year, resulting in the loss of 365 million dollars (Yang et al. 2019). In China, *Salmonella* infection cases are also frequently reported and accounted for 70–80% of bacterial food poisonings (Wang et al. 2006; Yang et al. 2016). Chen et al. reported that there were 134 outbreaks of food poisoning events caused by *Salmonella* in Guangxi from 1981 to 2003, which caused 7285 cases of salmonellosis (Chen et al. 2004). Between July 2010 and December 2011, nontyphoidal *Salmonella* were isolated from 316 (17.2%) of 1833 cases of acute gastroenteritis in children in Shanghai (Li et al. 2014a). After being infected with *Salmonella*, human and livestock can be asymptomatic carriers, which can reduce the fertility, aggravate the morbidity or mortality, and even be manifested as clinically fatal diseases. Pigs are considered to be one of the most important reservoir for many serovars of *Salmonella*, and most human infections are attributed to consumption of contaminated pork (Eurosurveillance editorial team 2012; Li et al. 2013; Vo et al. 2006a). Information on the distribution of different *Salmonella enterica* serovars in contaminated pork is important to public health.

Slaughterhouse is a main place where livestock and poultry products might be contaminated with *Salmonella*. In order to control the transmission of *salmonella*, the European Union has carried out many studies on pig slaughterhouses and processes. From these studies, Swart et al. have developed a model to evaluate the effects of different interventions (Swart et al. 2016). The swine herd population and pork production in China account for half of the world (Windhorst 2012). Therefore, it is of great public health significance to study the status of *Salmonella* contamination from pig slaughterhouses in China (Botteldoorn et al. 2004). As a big pork producer, China must strengthen its control of *Salmonella* transmission.

As of 2013, the total antibiotics usage in China was approximately 162,200 tons, of which 84,100 tons were for animals (Tang et al. 2016). The use of antibiotics in China accounts for about half of the world. These data show that China is one of the countries with the most severe abuse of antibiotics. In animals, salmonellosis is mainly treated with antibiotics for prevention and control. However, due to the abuse of antibiotics in recent years, the rate of *Salmonella* with drug resistance and even multidrug resistance has risen significantly resulting in the increased frequency of treatment failure in human clinical medicine (Hidalgo-Vila et al. 2008; Kariuki et al. 2015; Kingsley et al. 2009; Dahshan et al. 2011; Beyene et al. 2011; Hendriksen et al. 2009; Pan et al. 2009; Li and Liu 2005). *Salmonella* with antibiotic resistance in contaminated products could infect humans directly or transmit their resistance genes to human pathogens through the food chain, leading to the failure of antibiotic treatment and posing a threat to human health. Thus it is a reason that the disease caused by *Salmonella* is not well controlled clinically.

In this study, a total of 459 swine samples were randomly collected from a pig slaughterhouse between 2016 and 2017 in Yanzhou, China. All the isolates were examined for serotype distribution, antimicrobial resistance, major genotypes, and the relationship between drug resistance phenotype and drug resistance genes. The results could provide a reference for the epidemiological investigation of *Salmonella* in pigs.

## Materials and methods

### Sample collection and *Salmonella* isolation

During a period of 13 months, from October 2016 to October 2017, a total of 459 samples (distal ilea, n = 230; livers, n = 166; feces, n = 63) were collected at the start of the slaughter line from slaughtered pigs in a large scale industrialized slaughterhouse in Yangzhou, China. After collection, all samples were stored in sterilized containers with ice bags, and immediately processed to isolate *Salmonella* strains. Briefly, the samples of the ileum and liver were sheared, and 0.5 g fecal samples were picked. The samples mentioned above were added to 5 mL buffered peptone water (BPW; Neogen, Lansing, MI, USA) and incubated at 37 °C for 8–18 h. Subsequently, 100 μL pre-enriched culture was inoculated into in 5 mL of selenite cysteine (SC) broth at 37 °C for 12–18 h and then streaked on MacConkey and *Salmonella* and *Shigella* (SS) plates. After incubation for 18–24 h at 37 °C, suspected colonies were picked from MacConkey medium plates for purification. After purifying, the suspected *Salmonella* colonies were stained according to the Gram staining instruction manual. Then, the gram-negative bacteria were further confirmed by combined polymerase chain reaction (PCR) analysis using 2 pairs of primers [*Salmonella* enterotoxin gene (*stn*, 260 bp) and histidine transporter gene (*hut*, 495 bp)]. The wild-type *S*. Choleraesuis strain C78-3 (CVCC79103) was purchased from China Institute of Veterinary Drugs Control and used as a positive control. The primers used in this study are described in Table 1.

**Table 1:** Primers used for PCR amplification

Target genes	Nucleotide sequences	Size (bp)
*Salmonella* spp. detection primer sets		
*stn*-F	5′-CTTTGGTCGTAAAATAAGGCG-3′	260
*stn*-R	5′-TGCCCAAAGCAGAGAGATTC-3′	
*hut*-F	5′-ACTGGCGTTATCCCTTTCTCTGCTG-3′	495
*hut*-R	5′-ATGTTGTCCTGCCCCTGGTAAGAGA-3′	
16S rRNA primer set		
27F	5′-AGAGTTTGATCCTGGCTCAG-3′	1466
1492R	5′-TACGGTTACCTTGTTACGACTT-3′	
Antimicrobial resistance gene primer sets		
Quinolones		
* qnrA*-*F*	5′-TTCAGCAAGAGGATTTCTCA-3′	500
* qnrA*-*R*	5′-GGCAGCACTATTACTCCCAA -3′	
* qnrB*-*F*	5′-CCTGAGCGGCACTGAATTTT-3′	617
* qnrB*-*R*	5′-GTTTGCTGCTCGCCAGTCGA-3′	
* qnrC*-*F*	5′-GGGTTGTACATTTATTGAATC-3′	447
* qnrC*-*R*	5′-TCCACTTTACGAGGTTCT-3′	
* qnrD*-*F*	5′-TTACGGGGAATAGAGTTA-3′	468
* qnrD*-*R*	5′-AATCAGCCAAAGACCAAT-3′	
* qnrS*-*F*	5′-ACATAAAGACTTAAGTGATC-3′	619
* qnrS*-*R*	5′-CAATTAGTCAGGATAAAC-3′	
* qepA*-*F*	5′-CCAGCTCGGCAACTTGATAC-3′	570
* qepA*-*R*	5′-ATGCTCGCCTTCCAGAAAA-3′	
* oqxA*-*F*	5′-CTCGGCGCGATGATGCTC-3′	392
* oqxA*-*R*	5′-CACTCTTCACGGGAGACGA-3′	
* oqxB*-*F*	5′-TTCTCCCCCGGGGGGAAGTCCTCGGC-3′	512
* oqxB*-*R*	5′-CATTTTGGCGCGTA-3′	
Aminoglycosides		
* aac(6′)*-*Ib*-*F*	5′-TTGCGATGCTCTATGAGTGGCTA-3′	482
* aac(6′)*-*Ib*-*R*	5′-CTCGAATGCCTGGCGTGTT-3′	
* aadA1*-*F*	5′-GCGCCATCTCGAACCGACGTT-3′	573
* aadA1*-*R*	5′-GCCCAGTCGGCAGCGACATC-3′	
Sulfonamides		
* sul1*-*F*	5′-TGGCGTCGCGACTGCGAAAT-3′	813
* sul1*-*R*	5′-TGGTGACGGTGTTCGGCATTCT-3′	
* sul2*-*F*	5′-GTTTCTCCGATGGAGGCCGGT-3′	517
* sul2*-*R*	5′-AGCGAGGTTTCGGGAGCAGC-3′	
Trimethoprim		
* dfrA1*-*F*	5′-AGTGCCAAAGGTGAACAGCTCCT-3′	308
* dfrA1*-*R*	5′-ACATCACCTTCCGGCTCGATGTCT-3′	
β-lactamase		
* bla*_*OXA*-*1*_-*F*	5′-ATGAAAAACACAATACATATC-3′	830
* bla*_*OXA*-*1*_-*R*	5′-AATTTAGTGTGTTTAGAATGG-3′	
* bla*_*PSE*-*1*_-*F*	5′-CGCTTCCCGTTAACAAGTAC-3′	420
* bla*_*PSE*-*1*_-*R*	5′-CTGGTTCATTTCAGATAGCG-3′	
* bla*_*TEM*_-*F*	5′-ATAAAATTCTTGAAGACGAAA-3′	1080
* bla*_*TEM*_-*R*	5′-GACAGTTACCAATGCTTAATC-3′	
* Bla*_*CMY*-*2*_-*F*	5′-TGGCGGTTGCCGTTATCTAC-3′	210
* Bla*_*CMY*-*2*_-*R*	5′-CCCGTTTTATGCACCCATGA-3′	
Tetracyclines		
* tetA*-*F*	5′-TGGTCCGGAGGCCAGACGTG-3′	867
* tetA*-*R*	5′-TTCCGAGCATGAGTGCCCGC-3′	
* tetB*-*F*	5′-GGAGCTACTGGGGCTGTCGCACC-3′	374
* tetB*-*R*	5′-ACCCACACCGTTGCGGGAAT-3′	
* tetG*-*F*	5′-TCTTGCAGGAGCCGCAGTCGAT-3′	721
* tetG*-*R*	5′-GGCCGGCATGCCAACACCC-3′	
Chloramphenicols		
* catA1*-*F*	5′-TCTTGCCCGCCTGATGAATGC-3′	388
* catA1*-*R*	5′-AACCTGAATCGCCAGCGGCA-3′	
* floR*-*F*	5′-AACCCGCCCTCTGGATCAAGTCAA-3′	549
* floR*-*R*	5′-CAAATCACGGGCCACGCTGTATC-3′	
Virulence gene primer sets		
*mogA*-*F*	5′-ATTGGCTTAGTTTCTATCTCCG-3′	419
* mogA*-*R*	5′-CCTTCCAGCGTTTCTTTGA-3′	
* pvB*-*F*	5′-CCGTAGAGCAGACGCTGTAAGC-3′	1856
* spvB*-*R*	5′-GTATCTATGAGTTGAGTACCCCTATG-3′	
* spvC*-*F*	5′-CCGCAAAGTAGTGCATCTAAAC-3′	919
*spvC*-*R*	5′-CCATACTTACTCTGTCATCAAACG-3′	

### *Salmonella* serotyping

To evaluate the serotype distribution of the *Salmonella* isolates, the confirmed *Salmonella* strains were serotyped by slide agglutination test for O and H antigens of *Salmonella* using commercially available antiserum (Lanzhou Institute of Biological Products, China). Briefly, a single colony, picked with a loop, was evenly coated to a 10 μL of *Salmonella* standard antiserum on a slide. The slide was shaked gently for 1–2 min. The agglutinator which showed a uniform turbidity was determined as the serotype positive of *Salmonella* based on the measured antigenic formula according to GB/T 4789.4-2010. In this process, the negative control of saline group was also included.

### Analysis of the 16S rRNA sequence

To determine the homology of the *Salmonella* isolates, 16S rRNA genes of the identified strains were sequenced by Sangon Biotech. Primers for the amplification of 16S rRNA corresponding to the universal primers 27F and 1492R are listed in Table 1. The DNA sequences of 16S rRNA were edited and assembled using the programs SeqMan and Edit Seq (DNA Star, Laser Gene 6, Madison, WI, USA). The sequences were aligned using MEGA v6.0 and MegAlign software. Genetic distances were defined using the Kimura 2-parameter model (Kumar et al. 2004). The phylogenetic tree was constructed by the neighbour-joining method in MEGA v6.0 (Saitou et al. 1987). Percent divergence and similarity were calculated by comparing sequence pairs in relation by MegAlign.

### Antimicrobial susceptibility testing

Antimicrobial susceptibility test results of the *Salmonella* isolates to 22 kinds of antimicrobials was carried out in accordance with the standard Kirby-Bauer disk diffusion method recommended by the Clinical and Laboratory Standards Institute (CLSI 2017) (Berchieri et al. 2001). The reference strain, *Escherichia coli* ATCC 25922 was used as a control. The isolates were classified as susceptible, intermediate, or resistant according to the CLSI (2017) guidelines. *Salmonella* isolates resistant to at least 3 different antimicrobials were defined as multidrug resistance isolates (Pokharel et al. 2006). The following antibiotics were used in this study: ampicillin (AMP, 10 μg), mezlocillin (MEZ, 75 μg), amoxicillin/clavulanic acid (augmentin, AMC, 20/10 μg), cefoxitin (CFX, 30 μg), ceftriaxone (CRO, 30 μg), aztreonam (ATM, 30 μg), polymyxin B (POL, 300 IU), gentamicin (GEN, 10 μg), tobramycin (TOB, 10 μg), amikacin (AMK, 30 μg), kanamycin (KAN, 30 μg), neomycin (NEO, 30 μg), streptomycin (STR, 10 μg), tetracycline (TET, 30 μg), chloramphenicol (CHL, 30 μg), florfenicol (FFC, 30 μg), ciprofloxacin (CIP, 5 μg), enrofloxacin (ENR, 10 μg), sulfisoxazole (SUL, 300 μg), trimethoprim/sulfamethoxazole (bactrim, SXT, 1.25/23.75 μg), trimethoprim (TMP, 5 μg), and nitrofurantoin (NIT, 300 μg). The disks of 22 different antimicrobial agents were purchased from Hangzhou Microbiological Reagent Co., Ltd.

### PCR amplification of antimicrobial resistance genes

DNA templates of the *Salmonella* isolates for PCR were prepared according to the boiled lysis method (Ahmed et al. 2010). In short, all the *Salmonella* isolates were maintained in Luria–Bertani (LB) broth. Then, an overnight bacterial culture (200 μL) was mixed with 800 μL of distilled water and boiled for 10 min. The mixture samples were centrifuged at 4 °C for 5 min and the supernatant were used as the DNA templates. After extraction of DNA, the antimicrobial resistance genes, including quinolones, aminoglycosides, sulfonamides, trimethoprim, β-lactamase, tetracyclines, and chloramphenicols, were examined by PCR amplification, using the previously described primers listed in Table 1 (Petermann et al. 2011). The PCR products were subjected to electrophoresis in a 1.0% agarose gel, and sequenced by Sangon Biotech Co., Ltd. (Shanghai, China). The DNA sequences were compared with data in the GenBank database using the BLAST tool available at the National Center for Biotechnology Information website (http://www.ncbi.nlm.nih.gov).

### Detection of virulence genes

The *Salmonella* pathogenicity island-I (SPI-1) virulence gene *mogA* and the virulence genes *spvB* and *spvC* were selected to detect the virulence of *Salmonella* through the multiplex PCR as described by Skyberg et al. (Skyberg et al. 2006). The primers of virulence genes used in this study are described in Table 1.

## Results

### Isolation of *Salmonella*

To investigate the contamination status of *Salmonella* in a pig slaughterhouse, 459 samples (distal ilea, n = 230; livers, n = 166; feces, n = 63) were collected randomly from the slaughtered pigs in Yangzhou between October 2016 and October 2017. After enrichment, purification, Gram stain and PCR procedure, a total of 80 *Salmonella* isolates were recovered and identified from 459 (17.43%) samples. The isolation rate of *Salmonella* spp. was 27.83% (64/230) in ileum samples, 8.43% (14/166) in liver samples and 3.17% (2/63) in feces samples, respectively (Table 2). The isolates showed higher positive rate for *Salmonella* in ileum samples than that in liver and feces samples.

**Table 2:** Information of *Salmonella* isolated from a pig slaughterhouse in Yangzhou, China

No.	Serotype	Origin	Virulence genes			Resistance genes	Date
			*mogA*	*spvB*	*spvC*		
1	*S.* Sinstorf	Feces	−	−	−	*oqxA, oqxB, aac(6′)*-*Ib, sul1, sul2, tetA, tetG, catA1, floR*	2 Oct 2016
2	*S.* Typhimurium	Liver	+	+	+	*blapsE*-*1, tetG, catA1*	2 Oct 2016
3	*S.* Typhimurium	Ileum	+	+	+	*blapsE*-*1, tetG, catA1*	2 Oct 2016
4	*S.* Typhimurium	Ileum	+	−	−	*qnrS, oqxB, blatem, tetA, floR, catA1*	2 Oct 2016
5	*S.* Rissen	Ileum	+	−	−	*qepA, sul2, tetA*	2 Oct 2016
6	*S.* Derby	Liver	+	−	−	*qepA, oqxB, aac(6′)*-*Ib, blaoxA*-*1, tetA, tetG, floR, dfrA1*	22 Oct 2016
7	*S.* Sinstorf	Liver	+	−	−	*oqxA, aac(6′)*-*Ib, blaCMY*-*2, tetA, tetG, floR, dfrA1*	23 Oct 2016
8	*S.* Rissen	Ileum	+	−	−	*qepA, oqxB, blatem, tetA, catA1, dfrA1*	23 Oct 2016
9	*S.* Sinstorf	Liver	−	−	−	*oqxA, oqxB, aac(6′)*-*Ib, tetA, tetG, catA1, floR, dfrA1*	23 Oct 2016
10	*S.* Derby	Liver	+	−	−	*qepA, aac(6′)*-*Ib, blaoxA*-*1, tetA, dfrA1*	23 Oct 2016
11	*S.* Derby	Liver	+	−	−	*oqxA, oqxB, tetA, dfrA1*	4 Dec 2016
12	*S.* Newlands	Ileum	−	−	−	*tetA*	4 Dec 2016
13	*S.* Newlands	Ileum	−	−	−		4 Dec 2016
14	*S.* Newlands	Ileum	−	−	−	*catA1*	4 Dec 2016
15	*S.* Derby	Ileum	+	−	−	*aac(6′)*-*Ib, blatem, aadA1, floR*	4 Dec 2016
16	*S.* Derby	Ileum	+	−	−	*aac(6′)*-*Ib, aadA1*	4 Dec 2016
17	*S.* Derby	Ileum	+	−	−	*aac(6′)*-*Ib, aadA1*	10 Dec 2016
18	*S.* Typhimurium	Ileum	+	−	−	*qnrB, oqxA, oqxB, tetB, floR, catA1, aadA1*	10 Dec 2016
19	*S.* Derby	Ileum	+	−	−	*aac(6′)*-*Ib*	10 Dec 2016
20	*S.* Derby	Ileum	+	−	−		10 Dec 2016
21	*S.* Derby	Ileum	+	−	−	*tetA*	10 Dec 2016
22	*S.* Newlands	Ileum	−	−	−	*tetA*	10 Dec 2016
23	*S.* Newlands	Ileum	−	−	−		10 Dec 2016
24	*S.* Derby	Liver	+	−	−	*tetA*	26 Dec 2016
25	*S.* Newlands	Feces	−	−	−	*dfrA1*	26 Dec 2016
26	*S.* Derby	Ileum	+	−	−	*oqxA, aac(6′)*-*Ib, sul1, sul2, blaoxA*-*1, tetA, aadA1, floR*	26 Dec 2016
27	*S.* Typhimurium	Ileum	+	−	−	*qnrS, oqxB, blatem, tetA, floR, catA1*	26 Dec 2016
28	*S.* Rissen	Ileum	+	−	−	*tetA, aadA1, catA1*	26 Dec 2016
29	*S.* Typhimurium	Ileum	+	−	−	*qnrS, oqxB, blatem, tetA, floR, catA1*	26 Dec 2016
30	*S.* Derby	Liver	+	−	−	*oqxA, sul2, blatem, tetA, aadA1, catA1, dfrA1*	14 Jan 2017
31	*S.* Derby	Liver	+	−	−	*oqxA, sul2, blatem, tetA, aadA1, dfrA1*	14 Jan 2017
32	*S.* Derby	Liver	+	−	−	*oqxA, tetA, aadA1, dfrA1*	14 Jan 2017
33	*S.* Newlands	Ileum	−	−	−	*sul2, blapsE*-*1, tetA, catA1, floR*	14 Jan 2017
34	*S.* Rissen	Ileum	−	−	−	*sul2, catA1*	25 Feb 2017
35	*S.* Newlands	Ileum	−	−	−	*sul2, tetA, aadA1, floR*	25 Feb 2017
36	*S.* Derby	Ileum	+	−	−	*oqxA, aac(6′)*-*Ib, sul1, sul2, blaoxA*-*1, blapsE*-*1, tetA, aadA1, floR*	25 Feb 2017
37	*S.* Nchanga	Ileum	−	−	−	*sul2, blapsE*-*1, catA1, floR*	25 Feb 2017
38	*S.* Derby	Ileum	+	−	−	*oqxA, sul1, sul2, blaoxA*-*1, blapsE*-*1, tetA, aadA1, floR*	25 Feb 2017
39	*S.* Derby	Ileum	+	−	−	*oqxA, aac(6′)*-*Ib, sul1, sul2, blaoxA*-*1, tetA, aadA1, floR*	25 Feb 2017
40	*S.* Derby	Ileum	+	−	−	*oqxA, aac(6′)*-*Ib, sul1, sul2, blaoxA*-*1, tetA, aadA1, floR*	25 Feb 2017
41	*S.* Newlands	Ileum	−	−	−		25 Feb 2017
42	*S.* Derby	Liver	+	−	−	*aac(6′)*-*Ib, blaoxA*-*1, tetA*	2 Apr 2017
43	*S.* Newlands	Ileum	+	−	−	*sul2, tetA, aadA1, floR*	2 Apr 2017
44	*S.* Derby	Liver	+	−	−	*tetA, aadA1, catA1*	22 Apr 2017
45	*S.* Derby	Ileum	+	−	−	*aadA1*	22 Apr 2017
46	*S.* Derby	Ileum	+	−	−	*tetA*	22 Apr 2017
47	*S.* Newlands	Ileum	−	−	−		22 Apr 2017
48	*S.* Rissen	Ileum	+	−	−	*sul2, blatem, tetA, aadA1, catA1*	22 Apr 2017
49	*S.* Rissen	Ileum	+	−	−	*Blatem, tetA, aadA1, catA1*	22 Apr 2017
50	*S.* Rissen	Ileum	+	−	−	*Blatem, tetA, aadA1, catA1*	22 Apr 2017
51	*S.* Rissen	Ileum	+	−	−	*Blatem, tetA, aadA1, catA1*	22 Apr 2017
52	*S.* Rissen	Ileum	+	−	−	*Blatem, tetA, aadA1, catA1*	22 Apr 2017
53	*S.* Derby	Ileum	+	−	−	*Blatem, tetA, aadA1, catA1, floR*	22 Apr 2017
54	*S.* Rissen	Ileum	+	−	−	*Blatem, tetA, aadA1, catA1, dfrA1*	22 Apr 2017
55	*S.* Rissen	Ileum	+	−	−	*Blatem, tetA, aadA1, catA1*	22 Apr 2017
56	*S.* Rissen	Ileum	+	−	−	*Blatem, tetA, aadA1, catA1*	22 Apr 2017
57	*S.* Rissen	Ileum	+	−	−	*Blatem, tetA, aadA1, catA1*	22 Apr 2017
58	*S.* Rissen	Ileum	+	−	−	*sul2, blatem, tetA, aadA1, catA1, dfrA1*	22 Apr 2017
59	*S.* Derby	Ileum	+	−	−	*sul2, tetA, aadA1, catA1*	22 Apr 2017
60	*S.* Derby	Liver	+	−	−	*tetA, aadA1, catA1, floR*	20 May 2017
61	*S.* Derby	Ileum	+	−	−	*tetA*	20 May 2017
62	*S.* Rissen	Ileum	+	−	−	*qnrS, blatem, tetA*	20 May 2017
63	Untyped	Ileum	+	−	−	*blatem, tetA*	20 May 2017
64	*S.* Derby	Ileum	+	−	−	*tetA*	17 Jul 2017
65	*S.* Nchanga	Ileum	+	−	−	*aac(6′)*-*Ib, tetA*	17 Jul 2017
66	*S.* Derby	Ileum	+	−	−	*tetA*	20 Oct 2017
67	*S.* Derby	Ileum	+	−	−		20 Oct 2017
68	*S.* Derby	Ileum	+	−	−	*catA1*	20 Oct 2017
69	*S.* Derby	Ileum	+	−	−		20 Oct 2017
70	*S.* Derby	Liver	+	−	−		22 Oct 2017
71	*S.* Derby	Ileum	+	−	−		22 Oct 2017
72	*S.* Nchanga	Ileum	−	−	−	*qnrS, sul2, blapsE*-*1, tetA, aadA1, catA1, floR*	22 Oct 2017
73	*S.* Rissen	Ileum	+	−	−	*aadA1, catA1*	22 Oct 2017
74	*S.* Derby	Ileum	+	−	−		22 Oct 2017
75	*S.* Chester	Ileum	+	−	−	*tetB, catA1*	22 Oct 2017
76	*S.* Chester	Ileum	+	−	−	*tetB, catA1*	22 Oct 2017
77	*S.* Derby	Ileum	+	−	−	*oqxA, aac(6′)*-*Ib, sul1, sul2, blaoxA*-*1, blapsE*-*1, tetA, aadA1, catA1, floR*	22 Oct 2017
78	*S.* Typhimurium	Ileum	+	−	−	*sul2, tetB, catA1*	28 Oct 2017
79	*S.* Typhimurium	Ileum	+	−	−	*sul2, tetB, catA1*	28 Oct 2017
80	*S.* Typhimurium	Ileum	+	−	−	*sul2, tetB, catA1*	28 Oct 2017

### Serotyping of *Salmonella* isolates

Of the 80 isolates, 79 (98.75%) were typable and 1 (1.25%) was non-typable (Table 3). The 79 *Salmonella* isolates consisted of 7 serotypes: *S.* Derby, *S.* Rissen, *S.* Newlands, *S.* Typhimurium, *S.* Sinstorf, *S.* Nchanga, and *S.* Chester. *S.* Derby (35/80, 43.75%) was the predominant one, followed by *S.* Rissen (16/80, 20.00%) and *S.* Newlands (11/80, 13.75%). The isolates contained 3 serovar groups: B (46/80, 57.50%), C1 (16/80, 20.00%), and E1 (18/80, 22.5%).

**Table 3:** Serotype distribution of the *Salmonella* isolates

Groups of sero-group of *Salmonella*	Serotype of *Salmonella*	Numbers of isolates
Group B	*S*. Derby	35
	*S*. Typhimurium	9
	*S.* Chester	2
Group C1	*S*. Rissen	16
Group E1	*S*. Newlands	11
	*S*. Sinstorf	3
	*S*. Nchanga	3
	Untyped	1

### Phylogenetic analysis based on 16S rRNA gene sequences

**Fig. 1:**
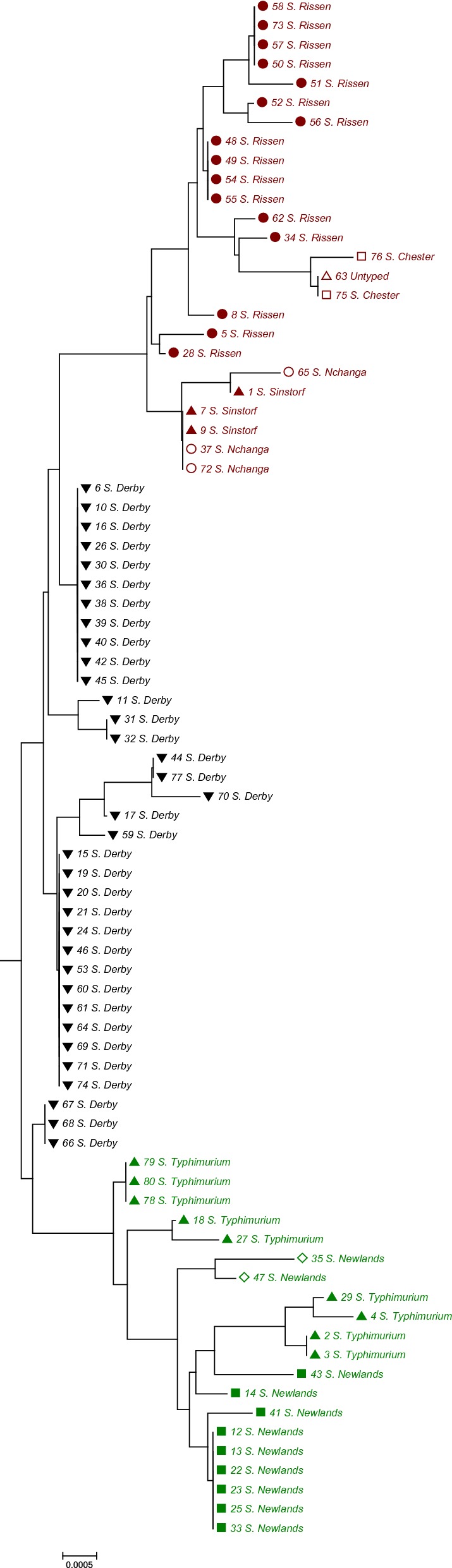
Phylogenetic tree of 80 *Salmonella* isolates based on 16S rRNA analysis. 16S rRNA gene sequences (1466 bp) were amplified by PCR and the nucleotide sequences determined. This is a neighbour-joining tree based on 80 *Salmonella* 16S rRNA sequences. The scale bar indicates one base substitution per 10,000 nt position. The number shown next to each node indicates the bootstrap value (1000 replicates)

To investigate the serotype homology of the *Salmonella* isolates, phylogenetic analysis of 16S rRNA sequences was carried out. All sequences of the isolates were submitted to GenBank (GenBank ID: MH548440–MH548519). A total of 80 16S rRNA gene sequences from isolates were compared based on differences in 16S rRNA sequence to construct an evolutionary tree. As shown in Fig. 1, the 80 isolates in the phylogenetic analysis were divided into three main clusters. We found that the same serotype can not be divided into discrete clusters, while the same cluster can contain multiple serotypes, which means that some *Salmonella* serotypes closely related to the gene sequences of 16S rRNA.

### Antimicrobial resistance of *Salmonella* isolates

All of the 80 *Salmonella* isolates were tested for antimicrobial susceptibility against 22 antimicrobial agents. The results of the antimicrobial resistance determination of the isolates are shown in Table 4. All the *Salmonella* isolates were susceptible to cefoxitin and amikacin and 65 (81.25%), 60 (75.00%), 55 (68.75%) and 54 (67.50%) isolates were resistant to tetracycline, ampicillin, bactrim and sulfisoxazole, respectively. Of particular note, all isolates were nonresistant against ceftriaxone, cefoxitin, azimium, polymyxin B, amikacin, and nitrofurantoin. In addition, 44 (55.00%), 31 (38.75%) and 30 (37.50%) of the isolates were moderately sensitive to enrofloxacin, polymyxin B and mezlocillin, respectively.

**Table 4:** Antimicrobial resistance rates of the 80 *Salmonella* isolates

Antimicrobials	Susceptible	Intermediate	Resistant
Ampicillin	6 (7.50%)	14 (17.50%)	60 (75.00%)
Mezlocillin	40 (50.00%)	30 (37.50%)	10 (12.50%)
Augmentin	52 (65.00%)	6 (7.50%)	22 (27.50%)
Ceftriaxone	68 (85.00%)	12 (15.00%)	0 (0.00%)
Cefoxitin	80 (100.00%)	0 (0.00%)	0 (0.00%)
Aztreonam	79 (98.75%)	1 (1.25%)	0 (0.00%)
Polymyxin B	49 (61.25%)	31 (38.75%)	0 (0.00%)
Gentamicin	64 (80.00%)	3 (3.75%)	13 (16.25%)
Tobramycin	65 (81.25%)	3 (3.75%)	12 (15.00%)
Amikacin	80 (100.00%)	0 (0.00%)	0 (0.00%)
Kanamycin	64 (80.00%)	4 (5.00%)	12 (15.00%)
Neomycin	70 (87.50%)	0 (0.00%)	10 (12.50%)
Streptomycin	31 (38.75%)	26 (32.50%)	23 (28.75%)
Tetracycline	14 (17.50%)	1 (1.25%)	65 (81.25%)
Florfenicol	46 (57.50%)	4 (5.00%)	30 (37.50%)
Ciprofloxacin	55 (68.75%)	17 (21.25%)	8 (10.00%)
Enrofloxacin	25 (31.25%)	44 (55.00%)	11 (13.75%)
Bactrim	25 (31.25%)	0 (0.00%)	55 (68.75%)
Sulfisoxazole	21 (26.25%)	5 (6.25%)	54 (67.50%)
Chloramphenicol	41 (51.25%)	2 (2.50%)	37 (46.25%)
Nitrofurantoin	79 (98.75%)	1 (1.25%)	0 (0.00%)
Trimethoprim	49 (61.25%)	7 (8.75%)	24 (30.00%)

The drug resistance profiles of the 80 isolates were constructed (Table 5). Among all of the 80 isolates, 73 (91.25%) of the isolates were resistant to at least one antibiotic, and 57 isolates showed multidrug resistance (resistant to three or more different antimicrobial agents), yielding the high rate of 71.25%. The *Salmonella* isolates in this study displayed a high and wide spectrum of antibiotic resistance. The most common resistance spectrums were AMP-STR-TET-SXT-SUL-TMP (n = 5) and AMP (n = 5). Totally, 46 resistance phenotypes of these isolates to 22 classes of antimicrobials were found in this study, among which most isolates were multidrug resistant to 6 and 8 classes of antimicrobials, accounted for 13.75% (11/80) and 12.50% (10/80), respectively. The highest drug resistance in the isolates were resistance to 14 antibacterial agents. Of particular note, our results showed that multidrug resistance of the isolates were frequently observed among the pig slaughterhouse.

**Table 5:** Antimicrobial resistance phenotypes of the 80 *Salmonella* isolates

Resistant phenotypes (numbers)	Numbers of isolates
AMP	5
STR	1
TET	3
FFC	1
AMP-TET	2
TET-SUL	2
TET-SXT	2
TET-SXT-SUL	1
AMP-TET-SXT	1
AMP-TET-SXT-SUL	2
STR-TET-SXT-TMP	1
TET-CHL-SXT-SUL	1
AMP-TET-SXT-SUL-TMP	4
AMP-MEZ-TET-SXT-SUL-TMP	1
AMP-AMC-TET-SXT	1
AMP-KAN-TET-CHL	2
AMP-AMC-TET-SXT-SUL	1
AMP-TET-CHL-SXT-SUL	1
AMP-MEZ-AMC-TET-SXT-SUL	1
AMP-STR-TET-SXT-SUL-TMP	5
AMP-TET-CHL-FFC-SXT-SUL	2
AMP-TET-CHL-SXT-SUL-TMP	1
STR-TET-CHL-FFC-SXT-SUL	1
AMP-MEZ-STR-TET-SXT-SUL-TMP	1
AMP-TET-CHL-FFC-SXT-SUL-TMP	1
AMP-AMC-TET-CHL-FFC-SXT-SUL	3
AMP-STR-TET-FFC-SXT-SUL-TMP	1
AMP-GEN-STR-TET-CHL-FFC-SXT-SUL	1
AMP-MEZ-AMC-TET-CHL-FFC-SXT-SUL	3
AMP-NEO-STR-TET-CHL-SXT-SUL-TMP	1
AMP-STR-TET-CHL-FFC-SXT-SUL-TMP	3
AMP-GEN-STR-TET-CHL-FFC-SXT-SUL-TMP	1
AMP-MEZ-STR-TET-CHL-FFC-SXT-SUL-TMP	1
AMP-AMC-GEN-TOB-KAN-STR-CHL-FFC-SXT-SUL	1
AMP-AMC-STR-TET-CHL-SXT-SUL	1
AMP-AMC-GEN-STR-TET-CHL-SXT-SUL	1
AMP-MEZ-AMC-TET-CHL-FFC-ENR-SUL	1
AMP-MEZ-GEN-TOB-STR-TET-CHL-FFC-CIP-ENR-SUL	1
AMP-GEN-TOB-KAN-NEO-STR-TET-CHL-FFC-CIP-ENR-SXT-SUL-TMP	1
AMP-AMC-GEN-TOB-STR-TET-CHL-ENR-SXT-SUL	1
AMP-AMC-TOB-KAN-NEO-TET-CHL-FFC-CIP-SXT-SUL	1
AMP-AMC-GEN-TOB-KAN-NEO-TET-CHL-FFC-ENR-SXT-SUL	1
AMP-AMC-GEN-TOB-KAN-NEO-TET-CHL-FFC-CIP-ENR-SXT-SUL	3
AMP-AMC-TOB-KAN-NEO-TET-CHL-FFC-CIP-ENR-SXT-SUL-TMP	1
AMP-MEZ-AMC-GEN-TOB-KAN-NEO-TET-CHL-FFC-ENR-SXT-SUL	1
AMP-AMC-GEN-TOB-KAN-NEO-TET-CHL-FFC-CIP-ENR-SXT-SUL-TMP	1

### Detection of antimicrobial resistance genes

Among the 22 resistance genes detected by PCR, 19 kinds of resistant genes in the *Salmonella* isolates were detected (Table 6). The highest rate was observed to the *tetA* gene (51/80, 63.75%), followed by *catA1* (38/80, 47.50%) and *aadA1* (33/80, 41.25%), which mediate the resistance to chloramphenicol and streptomycin, respectively. Only two isolates carried the *qnrB* gene and *blaCMY*-*2* gene, respectively, each accounting for 1.25%. It is noteworthy that all of the *aac*(*6*′)-*Ib* genes harbored the -*cr* mutation (Trp-Arg at locus 102 and Asp-Tyr at locus 179). Three antimicrobial resistance genes *qnrA*, *qnrC* and *qnrD* were not detected in the isolates. Moreover, 12.50% (10/80) of the isolates did not harbored any resistance genes, of which six of them exhibited extremely weak drug resistance (intermediate). We found that the resistance genes of tetracyclines (59/80, 73.75%) displayed the highest rate, which is in general consistency with the observations of antimicrobial resistance of the isolates. Higher rates of resistance genes to chloramphenicols (48/80, 60.00%) and aminoglycosides (41/80, 51.25%) than to sulfonamides (22/80, 27.50%) and trimethoprim (12/80, 15.00%) were observed. Among the 22 resistance genes, the genes including *tetA* (63.75%), *catA1* (47.50%), *aadA1* (41.25%) and *sul2* (26.25%) were the dominate genes in their corresponding resistance gene categories. Although the detection rates of quinolones and β-lactamase resistance genes were relatively high, the composition of each resistance gene was relatively dispersed.

**Table 6:** Antimicrobial resistance genes of the *Salmonella* isolates

Drug classes	Resistance genes	Number of isolates	Positive rates (%)
Quinolones 40.00%	*qnrA*	0	0.00
	*qnrB*	1	1.25
	*qnrC*	0	0.00
	*qnrD*	0	0.00
	*qnrS*	5	6.25
	*qepA*	4	5.00
	*oqxA*	14	17.50
	*oqxB*	9	11.25
Aminoglycosides 51.25%	*aac(6′)*-*Ib*	16	20.00
	*aadA1*	33	41.25
Sulfonamides 27.50%	*sul1*	8	10.00
	*sul2*	21	26.25
β-lactamase 43.75%	*bla*_*OXA*-*1*_	9	11.25
	*bla*_*PSE*-*1*_	8	10.00
	*bla*_*TEM*_	20	25.00
	*bla*_*CMY*-*2*_	1	1.25
Tetracyclines 73.75%	*tetA*	51	63.75
	*tetB*	6	7.50
	*tetG*	6	7.50
Chloramphenicols 60.00%	*catA1*	38	47.50
	*floR*	22	27.50
Trimethoprim 15.00%	*dfrA1*	12	15.00

### Relationship of antimicrobial resistance genes with antimicrobial susceptibility

The relationship between the antimicrobial resistance genes and the resistance phenotypes of the *Salmonella* isolates were analyzed by integrating the above data in this study. As shown in Table 7, the association of antimicrobial resistance genes with antimicrobial susceptibility was variable among differents *Salmonella* isolates. Among the six major categories of antimicrobials, the high relativity (> 80%) between the phenotypes and the antimicrobial resistance genotypes in the isolates was 33/41 (strains resistant to aminoglycosides antimicrobials/strains harbored resistant genes to aminoglycosides antimicrobials), 65/59 and 39/48, respectively for aminoglycosides, tetracyclines and chloramphenicols antimicrobials. In contrast, there were different in the quinolones, folate pathway inhibitors and β-lactamase with a coincidence rate of about 50%. For the specific antimicrobial resistance, the correlations between phenotypes and genotypes of the isolates for cephalosporins and tetracyclines were 0/1 and 65/59, respectively, much higher (> 90%) than other antimicrobials. These data indicated that the resistance to certain antimicrobials was associated, in part, with antimicrobial resistance genes.

**Table 7:** Resistance genes and phenotype relationship of *Salmonella* isolates

Drug classes	Quinolones	Aminoglycosides	Folate pathway inhibitors		β-lactamase		Tetracyclines	Chloramphenicols
	Ciprofloxacin Enrofloxacin	Gentamicin, Tobramycin Kanamycin, Neomycin Streptomycin	Sulfonamides	Trimethoprim	Penicillins	Cephalosporins	Tetracycline	Chloramphenicol Florfenicol
Resistance genes	*qnr*/*qepA*/*oqxA*/*oqxB*	*aadA1*/*aac(6′)*-*Ib*	*sul1*/*sul2*	*dfrA1*	*bla*_*OXA*-*1*_/*bla*_*PSE*-*1*_/*bla*_*TEM*_	*bla*_*CMY*-*2*_	*tetA*/*tetB*/*tetG*	*catA1*/*floR*
Number of isolates carrying drug-resistant genes	23	41	22	12	34	1	59	48
Number of drug-resistant isolates	12	33	59	24	60	0	65	39

### Detection of virulence genes

Among the 80 isolates, 3 virulence genes *mogA*, *spvB* and *spvC* were detected. We found that 81.25% (65/80) isolates at least carried the virulence gene of *mogA*, of which 2 *Salmonella* Typhimurium strains (2.50%) harbored the *mogA*, *spvB* and *spvC* virulence genes at the same time (Table 2). 18.75% (15/80) of the isolates did not carried any of the virulence genes. The results suggested that swine products in the slaughterhouse were commonly contaminated with *mogA* virulence gene. It is noteworthy that some isolates had the *spv* virulence genes, which is a great threat to public health safety.

## Discussion

*Salmonella* is one of the most common food-borne pathogens, with a wide range of hazards that can cause contamination of various agricultural products (Shao et al. 2011). It has been reported that the isolation rate of swine *Salmonella* in China was range from 11 to 35% and the predominant serotypes of the isolates were *S.* Derby, *S.* Typhimurium, *S.* Enteritidis, and *S.* Argona (Huang et al. 2012; Wang et al. 2016; Zou et al. 2012; Song et al. 2004; Kuang et al. 2015). *Salmonella* strains with strong pathogenicity were widespread, among them, *S.* Typhimurium and *S.* Enteritidis are the major non-typhoid *Salmonella* that cause diarrhea in humans. In this study, samples were collected randomly from the slaughtered pigs in mainland China between October 2016 and October 2017. Eventually, 80 *Salmonella* isolates were recovered from 459 samples of the pig slaughterhouse, and the overall isolation rate of *Salmonella* spp. was 17.43%. Among the isolates, the prevalence of *Salmonella* was 27.83% (64/230) in ileum samples, 8.43% (14/166) in liver samples and 3.17% (2/63) in feces samples, respectively. The prevalence of *Salmonella* from ileum was slightly higher than the 23.8% observed in Brazil (da Silva et al. 2012) and lower than the 36.5% reported in Huaian in China (Zhou et al. 2017).The serotyping results indicated that *S.* Derby (43.75%) in B group was the predominant serovar in the slaughterhouse and processing chain, which is consistent with previous studies (Bonardi et al. 2016; Piras et al. 2011). In fact, *S.* Derby has been shown to be the most common serovar across the world. For example, *S.* Derby has been shown to represent the most significant proportion of serovars in both pork and slaughterhouse in China (Cai et al. 2016; Li et al. 2014b). In general consistency with the reports by Zhou et al. (Zhou et al. 2017), we observed that *S.* Rissen was the second most common serovar in the slaughterhouse. A total of 14 *Salmonella* strains were isolated from liver samples consisting of *S.* Derby (n = 11), *S.* Chester (n = 2) and *S.* Typhimurium (n = 1). It is noteworthy that all the 3 serotypes of *Salmonella* have been reported to infect humans in China with the clinical syndromes including diarrhoea and septicemia (Liang et al. 2016; Zhou et al. 2013; Guo et al. 2015; Sun et al. 2014). These results suggest the importance of controlling *Salmonella* during slaughter process and regular surveillance for public health. In addition, *Salmonella* was isolated from October 2016 to October 2017, no significant difference in the prevalence was observed (data not shown).

It has been shown that *Salmonella* is widely drug-resistant and commonly multidrug resistant (Hidalgo-Vila et al. 2008; Li and Liu 2005; Chen et al. 2008; Wang et al. 2007, 2009). In this study, our results showed that 73 *Salmonella* isolates were resistant to at least one antimicrobial agent and most of the isolates showed multidrug resistance, mainly to tetracycline, ampicillin, bactrim, sulfisoxazole, and chloramphenicol. Our results concerning the phenomenon of particularly severe drug resistance are consistent with previously described findings in China (Yang et al. 2019; Lu et al. 2011). In this study, multidrug resistance isolate rate of *Salmonella* (71.25%) was similar to another two studies (71.4% and 73.9%) in China (Yang et al. 2019; Li et al. 2013). Our results showed that multidrug resistance of the isolates were frequently observed among the pig slaughterhouse. Reducing antibiotics use in pigs is particularly important to limit the emergence of multidrug resistance bacteria and to maintain good public health. According to previous reports (Chen et al. 2008; Vo et al. 2006b; Zhao et al. 2007), *Salmonella* strains were highly resistant to ampicillin, chloramphenicol, kanamycin, streptomycin, sulfonamides, tetracyclines and quinolones, consistent with the results in this study. Among the 80 *Salmonella* isolates, 7 isolates were none drug resistant strains including 5 *S.* Newlands strains, one *S.* Derby and one *S.* Typhimurium. The none drug resistant isolates were mainly distribution the *S.* Newlands, accounted for 45.45% (5/11) of all the isolated *S.* Newlands. Among all of the 9 *S.* Typhimurium isolates, one isolate was none drug resistant, and 5 isolates were only resistant to 1 or 2 kinds of antibiotics, indicating that the resistance of *S.* Newlands and *S.* Typhimurium were relatively low. Combined with the data of the resistant phenotypes and the antimicrobial resistance genes of the *Salmonella* isolates, the isolates have a high correlation between the phenotypes and genotypes of aminoglycosides, cephalosporins, tetracyclines and chloramphenicols, while the relationship between the resistance genes and the resistance phenotypes of the *Salmonella* isolates of trimethoprim, sulfonamides, penicillins and quinolones were relatively lower. These data indicated that the resistance to certain antimicrobials was associated with antimicrobial resistance genes. Moreover, the association of antimicrobial resistance genes with antimicrobial susceptibility were variable among differents *Salmonella* isolates. Some isolates harboring drug resistance genes were not highly drug-resistant while resistance genes could not be amplified by PCR from some highly drug-resistant isolate strains. It might be due to untested or unknown drug resistance genes in the resistant strains, and propose that further study is necessary.

*MogA* is a virulence gene associated with invasiveness on *Salmonella* SPI-1. *SpvB* gene has adenosine diphosphate (ADP) ribose transferase activity which mediates the modification of G-actin and the block of F-actin, and then disrupts the cytoskeletal function of actin (Tezcan-Merdol et al. 2005; Mesa-Pereira et al. 2013). The protein encoded by the *spvC* gene has a phosphorylated threonine lyase activity that inhibits MAP phosphokinase (Haneda et al. 2012; Mazurkiewicz et al. 2008). Notably, *spvB* and *spvC* are required for the expression of the *spv* gene simultaneously. The pathogenicity of *Salmonella* strains will greatly increase when both *spvB* and *spvC* genes exist at the same time. In this study, we found that swine products in the slaughterhouse were commonly contaminated with the *mogA* virulence gene (65/80, 81.25%). In general, the high detection rate of virulence genes highlights the pathogenic potential of these isolates, which may indicate serious salmonellosis and a threat to public health (Fardsanei et al. 2017). Our results also suggested that the *Salmonella* isolates harboring *spvB* and *spvC* virulence genes existed in the swine slaughterhouse, which is a great threat to public health.

The work described here highlights the prevalence and antimicrobial resistance of *Salmonella* in a pig slaughterhouse in mainland China. Swine products in the slaughterhouse were contaminated with multidrug resistant *Salmonella* commonly, even a small fraction of them might carry the *spv* virulence genes, which suggests efficient measures to facilitate the reasonable use of antimicrobials in animal husbandry must be taken to control *Salmonella* during slaughter for public health, underlying strict hygiene method and HACCP (Hazard Analysis and Critical Control Points) management are vital for reducing cross-contamination. To reduce *Salmonella* contamination, several other interventions have proven successful. Moreover, a mature and healthy livestock system should be established to strictly control the environmental hygiene, carcass hygiene, drinking water and feed hygiene, as well as the supervision of the processing and circulation of animal products. We believe that it is necessary to extend more studies about practical interventions in pig slaughterhouses to control *Salmonella* in China. Collectively, nationwide regular surveillance is needed to screen any changes in antimicrobial resistance patterns in *Salmonella* isolates in the swine industry.

## Data Availability

Not applicable.
